# Supplementary material for: A confounder controlled machine learning approach: Group analysis and classification of schizophrenia and Alzheimer’s disease using resting-state functional network connectivity
Source: PLoS One. 2024 May 20;19(5):e0293053. doi: 10.1371/journal.pone.0293053 (PMC11104643; doi:10.1371/journal.pone.0293053)
Supplement: S1 Fig — (A-B) demonstrate that SZ subjects are considerably younger than AD subjects. (C) reveals that the male and female counts within a dataset are unequal, particularly in B-SNIP. (D) highlights that the number of sites in ADNI is significantly greater than in B-SNIP. (PDF) [file pone.0293053.s001.pdf]

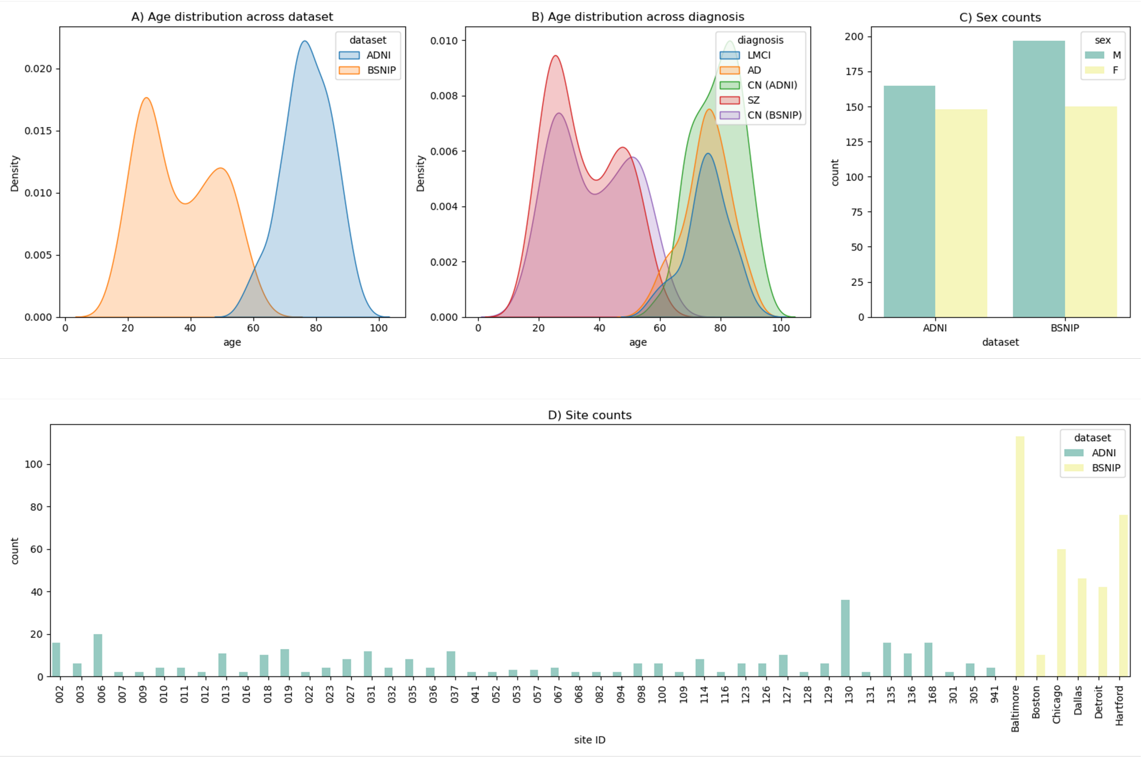

S1 Fig.: Distribution of age, gender, and site ID across datasets. (A-B) demonstrate that SZ subjects are considerably younger than AD subjects. (C) reveals that the male and female counts within a dataset are unequal, particularly in B-SNIP. (D) highlights that the number of sites in ADNI is significantly greater than in B-SNIP.
