# Supplementary material for: A confounder controlled machine learning approach: Group analysis and classification of schizophrenia and Alzheimer’s disease using resting-state functional network connectivity
Source: PLoS One. 2024 May 20;19(5):e0293053. doi: 10.1371/journal.pone.0293053 (PMC11104643; doi:10.1371/journal.pone.0293053)
Supplement: S2 Fig — (PDF) [file pone.0293053.s002.pdf]

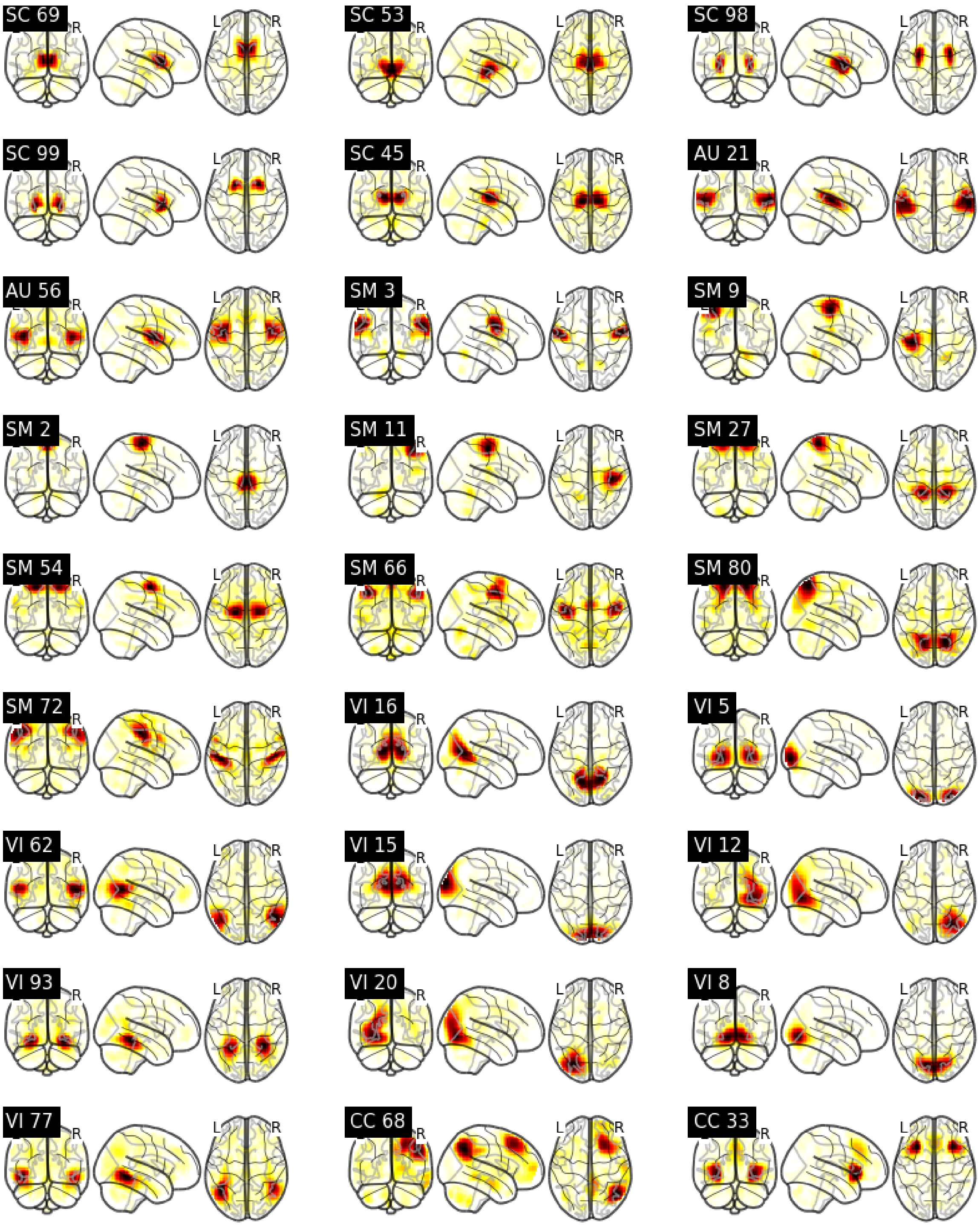

S2 Fig.: ICA-driven spatial maps, (the Neuromark templates).

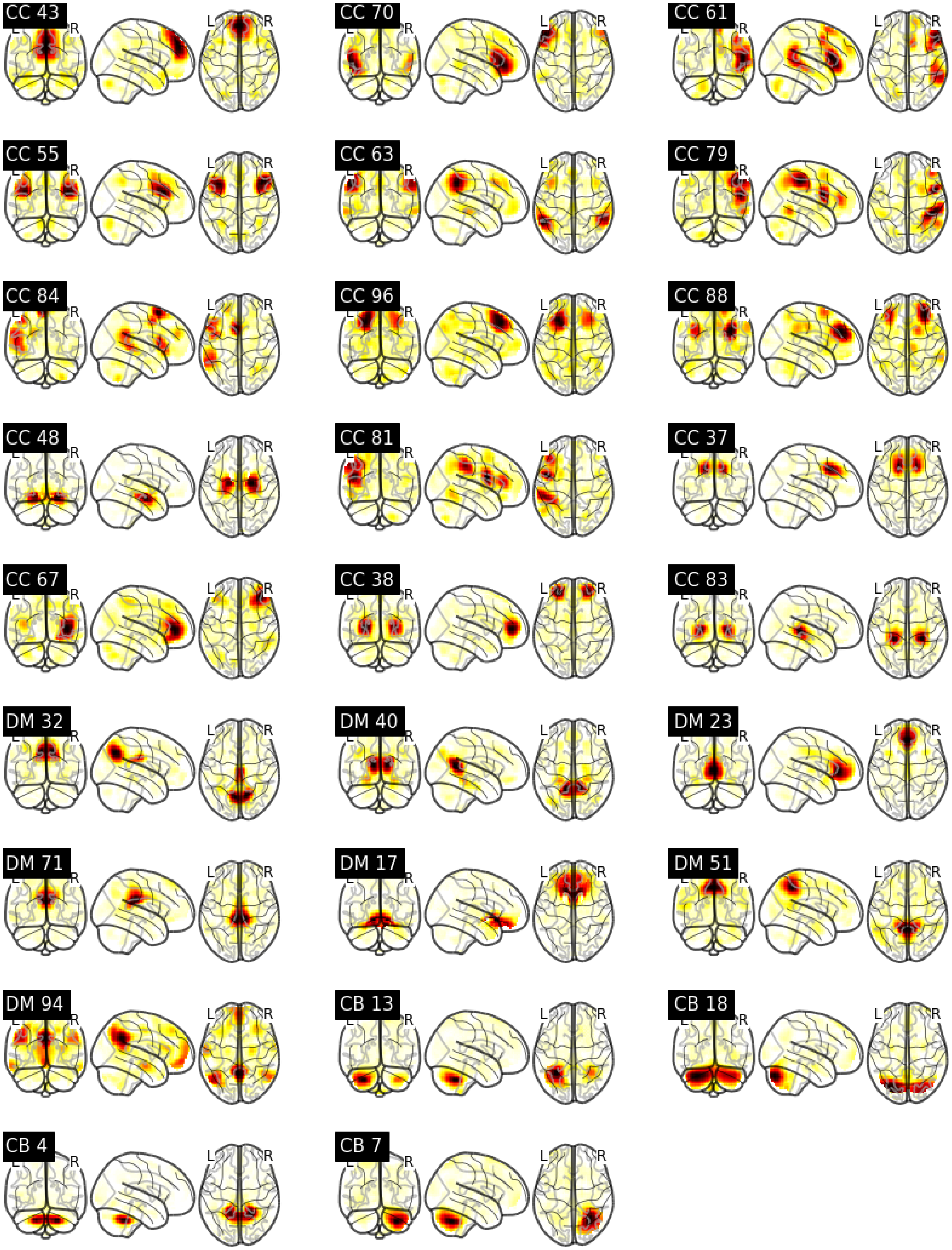

S2 Fig.: ICA-driven spatial maps, (the Neuromark templates, cont.).
