# Supplementary material for: A confounder controlled machine learning approach: Group analysis and classification of schizophrenia and Alzheimer’s disease using resting-state functional network connectivity
Source: PLoS One. 2024 May 20;19(5):e0293053. doi: 10.1371/journal.pone.0293053 (PMC11104643; doi:10.1371/journal.pone.0293053)
Supplement: S3 Fig — Comparing the results of AD vs. CN and SZ vs. CN to AD vs. CN:B-SNIP and SZ vs. CN:ADNI suggest that the ML and NN models are biased by the confounders. (PDF) [file pone.0293053.s003.pdf]

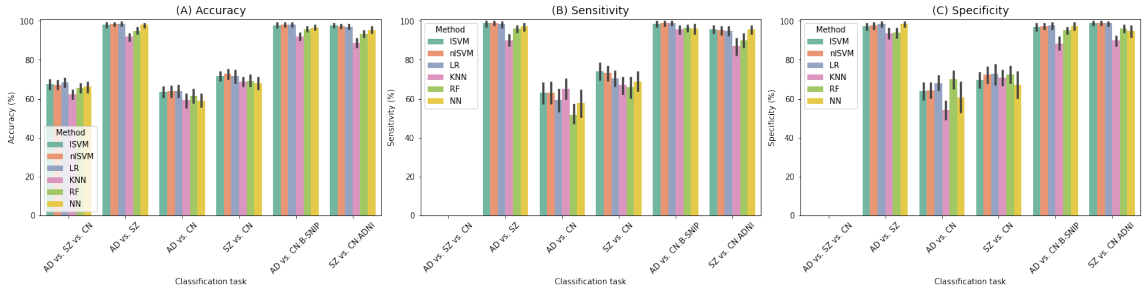

S3 Fig.: Accuracy, sensitivity, and specificity ($Mean \pm SE$) in the presence of confounders. Comparing the results of AD vs. CN and SZ vs. CN to AD vs. CN:B-SNIP and SZ vs. CN:ADNI suggest that the ML and NN models are biased by the confounders.
