# Supplementary material for: A confounder controlled machine learning approach: Group analysis and classification of schizophrenia and Alzheimer’s disease using resting-state functional network connectivity
Source: PLoS One. 2024 May 20;19(5):e0293053. doi: 10.1371/journal.pone.0293053 (PMC11104643; doi:10.1371/journal.pone.0293053)
Supplement: S1 Table — (PDF) [file pone.0293053.s004.pdf]

S1 Table: Control subject IDs used from ADNI dataset

IDs
002_S_0295, 002_S_0413, 002_S_0685, 002_S_1261, 002_S_1280, 002_S_4213, 002_S_4225, 002_S_4262, 003_S_4119, 003_S_4288, 003_S_4350, 006_S_0498, 006_S_0731, 006_S_4150, 006_S_4357, 006_S_4449, 006_S_4485, 006_S_6209, 006_S_6234, 006_S_6277, 006_S_6375, 007_S_1222, 009_S_0751, 010_S_0419, 010_S_4345, 011_S_0021, 011_S_4278, 012_S_4026, 013_S_4579, 013_S_4580, 013_S_4616, 013_S_4731, 013_S_6780, 016_S_4121, 018_S_4257, 018_S_4313, 018_S_4349, 018_S_4399, 018_S_4400, 019_S_4367, 019_S_4835, 019_S_6186, 022_S_6069, 023_S_0031, 023_S_1190, 027_S_0074, 027_S_0120, 027_S_6001, 027_S_6183, 031_S_4021, 031_S_4032, 031_S_4218, 031_S_4474, 031_S_4496, 032_S_0677, 032_S_1169, 035_S_0156, 035_S_0555, 035_S_4464, 035_S_6156, 036_S_4389, 036_S_6316, 037_S_0303, 037_S_0454, 037_S_4028, 037_S_4071, 037_S_4308, 037_S_4410, 041_S_4037, 052_S_6412, 053_S_4578, 057_S_0934, 067_S_0056, 067_S_0059, 068_S_0127, 082_S_4224, 094_S_4234, 098_S_0896, 098_S_4003, 098_S_4275, 100_S_0069, 100_S_1286, 100_S_4469, 109_S_4499, 114_S_0416, 114_S_6057, 114_S_6063, 114_S_6113, 116_S_0382, 123_S_0072, 123_S_0106, 123_S_0298, 126_S_0680, 126_S_6559, 127_S_0259, 127_S_4148, 127_S_4198, 127_S_4604, 127_S_4645, 128_S_0272, 129_S_0778, 129_S_4369, 129_S_4371, 130_S_0969, 130_S_4343, 130_S_4352, 130_S_6019, 130_S_6027, 130_S_6035, 130_S_6037, 130_S_6043, 130_S_6105, 130_S_6111, 130_S_6137, 130_S_6161, 130_S_6319, 130_S_6361, 130_S_6372, 130_S_6388, 130_S_6390, 130_S_6391, 131_S_6170, 135_S_4446, 135_S_4598, 135_S_6104, 135_S_6359, 135_S_6360, 135_S_6411, 135_S_6473, 135_S_6509, 136_S_0186, 136_S_4269, 136_S_4433, 136_S_4726, 136_S_4727, 168_S_6049, 168_S_6051, 168_S_6059, 168_S_6062, 168_S_6064, 168_S_6065, 168_S_6085, 168_S_6086, 301_S_6224, 305_S_6157, 305_S_6188, 305_S_6313, 941_S_1195, 941_S_4100
