# Supplementary material for: A confounder controlled machine learning approach: Group analysis and classification of schizophrenia and Alzheimer’s disease using resting-state functional network connectivity
Source: PLoS One. 2024 May 20;19(5):e0293053. doi: 10.1371/journal.pone.0293053 (PMC11104643; doi:10.1371/journal.pone.0293053)
Supplement: S2 Table — (PDF) [file pone.0293053.s005.pdf]

S2 Table: Networks ID, domain, and region identified by ICA

ID	*Domain*	*Region*	*ID*	*Domain*	*Region*
IC 69	SC	Caudate	IC 68	CC	Inferior parietal lobule
IC 53	SC	Subthalamus/hypothalamus	IC 33	CC	Insula
IC 98	SC	Putamen	IC 43	CC	Superior medial frontal gyrus
IC 99	SC	Caudate	IC 70	CC	Inferior frontal gyrus
IC 45	SC	Thalamus	IC 61	CC	Right inferior frontal gyrus
IC 21	AU	Superior temporal gyrus	IC 55	CC	Middle frontal gyrus
IC 56	AU	Middle temporal gyrus	IC 63	CC	Inferior parietal lobule
IC 3	SM	Postcentral gyrus	IC 79	CC	Left inferior parietal lobule
IC 9	SM	Left postcentral gyrus	IC 84	CC	Supplementary motor area
IC 2	SM	Paracentral lobule	IC 96	CC	Superior frontal gyrus
IC 11	SM	Right postcentral gyrus	IC 88	CC	Middle frontal gyrus
IC 27	SM	Superior parietal lobule	IC 48	CC	Hippocampus
IC 54	SM	Paracentral lobule	IC 81	CC	Left inferior parietal lobule
IC 66	SM	Precentral gyrus	IC 37	CC	Middle cingulate cortex
IC 80	SM	Superior parietal lobule	IC 67	CC	Inferior frontal gyrus
IC 72	SM	Postcentral gyrus	IC 38	CC	Middle frontal gyrus
IC 16	VI	Calcarine gyrus	IC 83	CC	Hippocampus
IC 5	VI	Middle occipital gyrus	IC 32	DM	Precuneus
IC 62	VI	Middle temporal gyrus	IC 40	DM	Precuneus
IC 15	VI	Cuneus	IC 23	DM	Anterior cingulate cortex
IC 12	VI	Right middle occipital gyrus	IC 71	DM	Posterior cingulate cortex
IC 93	VI	Fusiform gyrus	IC 17	DM	Anterior cingulate cortex
IC 20	VI	Inferior occipital gyrus	IC 51	DM	Precuneus
IC 8	VI	Lingual gyrus	IC 94	DM	Posterior cingulate cortex
IC 77	VI	Middle temporal gyrus	IC 13	CB	Cerebellum
			IC 18	CB	Cerebellum
			IC 4	CB	Cerebellum
			IC 7	CB	Cerebellum
