# Supplementary material for: A confounder controlled machine learning approach: Group analysis and classification of schizophrenia and Alzheimer’s disease using resting-state functional network connectivity
Source: PLoS One. 2024 May 20;19(5):e0293053. doi: 10.1371/journal.pone.0293053 (PMC11104643; doi:10.1371/journal.pone.0293053)
Supplement: S3 Table — (PDF) [file pone.0293053.s006.pdf]

S3 Table: Hyperparameters of support vector machine (SVM)

kernel	***c***	***gamma***	***degree***	***coef0-range***
linear	0.0001, 0.001, 0.01, 0.1, 1, 10, 100	-	-	-
rbf	0.0001, 0.001, 0.01, 0.1, 1, 10, 100	scale, auto	-	-
poly	0.0001, 0.001, 0.01, 0.1, 1, 10, 100	scale, auto	0, 1, 2, 3, 4, 5, 6, 7, 8, 9	0.0, 0.1
sigmoid	0.0001, 0.001, 0.01, 0.1, 1, 10, 100	scale, auto	-	0.0, 0.1
precomputed	0.0001, 0.001, 0.01, 0.1, 1, 10, 100	-	-	-
