# Supplementary material for: A confounder controlled machine learning approach: Group analysis and classification of schizophrenia and Alzheimer’s disease using resting-state functional network connectivity
Source: PLoS One. 2024 May 20;19(5):e0293053. doi: 10.1371/journal.pone.0293053 (PMC11104643; doi:10.1371/journal.pone.0293053)
Supplement: S4 Table — (PDF) [file pone.0293053.s007.pdf]

**S4 Table:** Hyperparameters of logistic regression (LR)

| ***solver*** | ***c*** | ***penalty*** |
|---|---|---|
| newton-cg | 0.0001, 0.001, 0.01, 0.1, 1, 10, 100, 1000 | none, l2 |
| lbfgs | 0.0001, 0.001, 0.01, 0.1, 1, 10, 100, 1000 | none, l2 |
| liblinear | 0.0001, 0.001, 0.01, 0.1, 1, 10, 100, 1000 | l1, l2 |
| sag | 0.0001, 0.001, 0.01, 0.1, 1, 10, 100, 1000 | none, l2 |
| saga | 0.0001, 0.001, 0.01, 0.1, 1, 10, 100, 1000 | none, l1, l2 |
