# Supplementary material for: A confounder controlled machine learning approach: Group analysis and classification of schizophrenia and Alzheimer’s disease using resting-state functional network connectivity
Source: PLoS One. 2024 May 20;19(5):e0293053. doi: 10.1371/journal.pone.0293053 (PMC11104643; doi:10.1371/journal.pone.0293053)
Supplement: S5 Table — (PDF) [file pone.0293053.s008.pdf]

S5 Table: Hyperparameters of random forest (RF)

criterion	***n-neighbors***	***max-depth***	***min-samples-split***	***min-samples-leaf***
gini, entropy	100, 200, 300, 400, 500	1, 3, 5, 10, 20, 30, 40, 50, 100, None	1, 3, 5, 10, 20, 30, 40, 50, 100, None	1, 2, 4
