# Supplementary material for: A confounder controlled machine learning approach: Group analysis and classification of schizophrenia and Alzheimer’s disease using resting-state functional network connectivity
Source: PLoS One. 2024 May 20;19(5):e0293053. doi: 10.1371/journal.pone.0293053 (PMC11104643; doi:10.1371/journal.pone.0293053)
Supplement: S6 Table — (PDF) [file pone.0293053.s009.pdf]

S6 Table: Hyperparameters of k-nearest neighbor (KNN)

algorithm	***n-neighbors***	***weights***	***leaf-size***	***p***
auto, ball-tree, kd-tree, brute	1, 2, 3, 4, 5, 6, 7, 8, 9, 10, 20, 30, 40	uniform, distance	1, 2, 3, 4, 5, 10, 20, 30, 40, 50	1, 2
