# Supplementary material for: A confounder controlled machine learning approach: Group analysis and classification of schizophrenia and Alzheimer’s disease using resting-state functional network connectivity
Source: PLoS One. 2024 May 20;19(5):e0293053. doi: 10.1371/journal.pone.0293053 (PMC11104643; doi:10.1371/journal.pone.0293053)
Supplement: S7 Table — (PDF) [file pone.0293053.s010.pdf]

S7 Table: Hyperparameters of neural network (NN)

learning rate	***batch size***	***hidden layer size***	***optimizer***
0.01, 0.001, 0.0001	4, 8, 16, 32, 64	4, 8, 16, 32	Adam
